# Microscopy of the Dental Tissues. New Series

**Published:** 1868-12

**Authors:** S. P. Cutler


### AMERICAN JOURNAL

OF

### DENTAL SCIENCE.

Vol. 11. THIRD SERIES--DECEMBER, 1868. No. 8.

### ARTICLE I.

### *Microscopy of the Dental Tissues. New Series.*

By S. P. Cutler M.D., A.E.G., D.D.S.,

*Continued.*

Histology and Physiology—Occlusion of Alveolus after the Extraction of Teeth.

To return to the alveolus. The acid theory, as advanced by myself, in relation to bone absorption, is strikingly sustained by the decay of teeth, which old dentists agree is chiefly the result of local acid action, acting on the decaying portion of a tooth from an acid condition of the saliva generated in the mouth and its glands. This acid may be exceedingly weak, so much so as scarcely to give an acid reaction with litmus, still this acid is sufficiently active to disintegrate the phosphate of lime of the tooth, it having a stronger affinity for the lime than the phosphoric acid. In the crown form of dental caries, we notice a disintegration of the lime for a considerable depth into the tooth, before the osteione, or what I have called dentine, has given way at all, still retaining all the tubular structures quite perfect, only presenting a worm-eaten crowded appearance in some of the tubuli.

I have maintained that waste or absorption in the fleshy tissues was the result of oxidation, which has been given in a former article. I also maintain that the osteine of bones is carried away by oxidation, the same as any other soft tissue, after the lime is disintegrated by acid action, one keeping pace with the other, or a paripassee movement, even in a state of perfect health. In the case of a decayed tooth, as above stated, the dentine is not oxidised away, even in no case, as fast as the lime is removed. This would, at first sight, seem to militate against the hypothesis advanced by myself, but this is really not the case at all, as the decay of a tooth is dependent chiefly on local outside action, independent of the constitution, though not wholly so. We find in some cases, that the adherent dentine possesses sensibility, and has the tubular nerve fibrils intact. There are other forms of dental decay, where the dentine is removed nearly as fast as the earthy salts, in such cases I maintain the animal portion is removed by local oxidation, mainly because it is carried on beyond the point of vital control. All septemic dental changes, such as softening of teeth, is the work of an acid and all waste in other bones, either normal or abnormal, so far as the earthy portions are concerned, as heretofore given. No other hypothesis is in any way satisfactory or conclusive. The above fully reconciles our hitherto doubts and uncertainties, on an unsettled question in my humble judgment. It also accounts for the restoration of broken or injured bones, for the hardening of bones in old age, and for the softening of bones in certain diseases, as Mollites Ossium or Osteo Malacie, where the lime is entirely removed, leaving only the gelatinous or cartilaginous portion of the one behind. In all such cases the acid diathesis has removed the earth to an abnormal degree, and all fresh or nutrient phosphate of lime being carried or supplied to the bones by the constitution, are also carried out again by an excess of acid, hence the normal nutrition of the bone or bones affected, fail to benefit thereby, the cartilage remaining is nutrified and wasted, perhaps now more rapidly than before the bone became affected. Such bones are, in some cases, susceptible of cure by constitutional remedies, as these are constitutional diseases. To return to the subject of animal temperature. It is maintained by Leibig, Lehman, and all other eminent modern chemists, that animal temperature is sustained by all chemical and vital processes going on in the organism. This is true, though the precise and definite *modus operandi* has not been satisfactory settled as yet, hence I am justified in advancing the ideas given in my former articles.

It would seem wonderful and unaccountable, that the temperature of warm blooded animals should be so constant and persistent against the constant and ever varying outside temperature, or thermal changes in some climates having a range of 135° degrees, or thereabouts, during 12 months. Still the temperature of well fed animals and man, with proper exercise and habits, will not vary so much as one degree in the interior of the body, at the same time there is taken into the interior of the chest 26 cubic inches of the outside ever varying atmosphere every tidal respiration, and 18 such respirations per minute, gives 468 cubic inches per minute coming in contact with the circulating blood, spread over and around 600,000 air vesicles, with a superfices in man of 1400 square feet.

This immense aerating surface, constantly exposed, as it is, to the changeable air, would naturally, by radiation, undergo similar changes, were it not for the forces going on in every part of the organism to counteract such influence. In cold blooded animals, similar changes follow the surrounding medium, as both vital and chemical are much slower and less resistant and persistent and change with atmospherical and aqueous changes, their temperature generally only about 2° above the surrounding temperature, hence a snake will live six months on one frog, without growing lean.

But to return to warm blooded animals. All changes of whatever nature, either vital or chemical, that takes place in the organism is a thermal disturber, that is, all condensations give up latent heat and all expansions absorb latent heat; that is to say, if a gas combines so as to form by its combination a liquid or solid, that gas gives up its caloric of gasidity or elasticity, which caloric so set free becomes free heat subject to be absorbed by the surrounding tissues.

Any body such as a solid becoming liquid has to receive additional caloric from some other source in order to undergo such change, so when a liquid is converted into gas or vapor such liquid has to absorb a certain amount of caloric from some other source before such change can take place. Now reverse the order and the reverse takes place, that is to say, when a gas is condensed to a liquid state, that gas has to give up a certain amount of caloric to become liquid or acid by combining with some other body resulting in a new gaseous body *compound*, whereby the volume of the former gas underwent condensation, a certain amount of heat must be set free and become free heat. Also when a liquid becomes a solid the volume becomes lessened and a certain amount of heat of liquidity has to be parted with to form a solid body. Different solids, liquids and gases all differ in their relative amounts of specific heat that controls their respective forms. In all ordinary oxygen combustions *which are oxidations*, such as burning of coal or wood, tallow or gas or rusting of iron, same as oxidation of animal tissues, the oxygen is the sole source of heat, none whatever comes from the carbon or hydrogen. In the combustible bodies the latent heat is forced from the oxygen by the chemical union and set free, which becomes free or sensible heat, such as we see and feel in our grates and fire places. Even in the burning of gas a certain condensation of oxygen takes place, the hydrogen and carbon of the gas condense and give up some of their caloric to form water and carbonic acid, which originally came from the oxygen where the gas was made from the combustion of the coal that heat up the retorts to distil over the gas residual in the coal, which was in the form of a solid. Now the same thing takes place in the animal organism by cell and all other oxidation, such as that which takes place in all fluids that contain hydrocarbons, as in the lymph and liquor sanguinis and the free cells of the circulation and lymph.

It will be safe to state that all the heat generated in the organism known as animal heat, comes exclusively from oxygen, *free oxygen*, taken from without and none from the oxygen already combined in the tissue in a semi-solid state; on the contrary, a certain amount of caloric has to be absorbed from the condensing oxygen to form carbonic acid and water by cell and all other oxidations taking place, resulting in animal or vital heat which is converted into life or vital force.

The capacity of all bodies for caloric, is diminished as bodies condense or become lessened in volume, and, *vice versa*, the capacities for caloric are increased as bodies expand, hence bodies to expand from a denser to a rarer state, absorb caloric in order to suffer such change and *vice versa*, as above repeated. This may be explained on the principle of saturating a sponge, it expands and absorbs water, and by compression the capacity for the liquid being lessened the water is forced out. As the solid impenetrable atoms of matter according to Dutton's theory are brought closer together there cannot be as much room for caloric as when farther removed, supposing every atom to be surrounded by the force known as caloric heat. The Daltonian theory now is discarded by many eminent writers, though the newer theories do not give as satisfactory explanations, hence I allude to the former. I maintain that matter and force are inseparable, the amount of atoms in a given body are the same, while the amount of caloric or force is ever varying and governed by surrounding nuclei and that the relative distances of solid atoms are ever varying, the wider apart the more room for accumulation of forces or modification of the same force whatever it may be.

*To be Continued.*

